# Expression of a pathogenic mutation of SOD1 sensitizes aprataxin-deficient cells and mice to oxidative stress and triggers hallmarks of premature ageing

**DOI:** 10.1093/hmg/ddu500

**Published:** 2014-09-30

**Authors:** Jean Carroll, Tristan K.W. Page, Shih-Chieh Chiang, Bernadett Kalmar, David Bode, Linda Greensmith, Peter J Mckinnon, Julian R. Thorpe, Majid Hafezparast, Sherif F. El-Khamisy

**Affiliations:** 1 Genome Damage and Stability Center, University of Sussex, Brighton BN1 9RQ, UK; 2 School of Life Science, University of Sussex, Brighton BN1 9QG, UK; 3 Department of Molecular Biology and Biotechnology, Krebs Institute, University of Sheffield, Sheffield S10 2TN, UK; 4 Sobell Department of Motor Neuroscience and Movement Disorders, UCL Institute of Neurology, Queen Square, London WC1N 3BG, UK; 5 Department of Genetics, St Jude Children's Research Hospital, Memphis, TN 38105-3678, USA and; 6 Center of Genomics, Helmy Institute for Medical Sciences, Zewail City of Science and Technology, Giza, Egypt

## Abstract

Aprataxin (APTX) deficiency causes progressive cerebellar degeneration, ataxia and oculomotor apraxia in man. Cell free assays and crystal structure studies demonstrate a role for APTX in resolving 5′-adenylated nucleic acid breaks, however, APTX function in vertebrates remains unclear due to the lack of an appropriate model system. Here, we generated a murine model in which a pathogenic mutant of superoxide dismutase 1 (SOD1^G93A^) is expressed in an *Aptx*−/− mouse strain. We report a delayed population doubling and accelerated senescence in *Aptx*−/− primary mouse fibroblasts, which is not due to detectable telomere instability or cell cycle deregulation but is associated with a reduction in transcription recovery following oxidative stress. Expression of SOD1^G93A^ uncovers a survival defect *ex vivo* in cultured cells and *in vivo* in tissues lacking Aptx. The surviving neurons feature numerous and deep nuclear envelope invaginations, a hallmark of cellular stress. Furthermore, they possess an elevated number of high-density nuclear regions and a concomitant increase in histone H3 K9 trimethylation, hallmarks of silenced chromatin. Finally, the accelerated cellular senescence was also observed at the organismal level as shown by down-regulation of insulin-like growth factor 1 (IGF-1), a hallmark of premature ageing. Together, this study demonstrates a protective role of Aptx *in vivo* and suggests that its loss results in progressive accumulation of DNA breaks in the nervous system, triggering hallmarks of premature ageing, systemically.

## INTRODUCTION

Aprataxin (APTX) belongs to the histidine triad domain superfamily of nucleotide hydrolases/transferases with preference at removing AMP from DNA and from DNA/RNA junctions (1,2). AMP-linked DNA structures are normal intermediates of DNA ligation that are predicted to accumulate during premature ligation cycles. Premature ligation can occur if the ligase attempts to ligate DNA breaks before 3′-end processing has occurred. Co-deletion of APTX together with 3′-end processing enzymes results in synergistic repair defects (3,4). Premature ligation can also occur during the process of ribonucleotide removal from genomic DNA (2). APTX appears to possess both nuclear and nucleolar functions since it associates with components of the nuclear single- and double-strand break repair machinery such as XRCC1 and XRCC4, and with nucleolar proteins such as nucleolin, and nucleophosmin (5,6). APTX has also been reported to localize to mitochondria and maintain mitochondrial DNA integrity (7). Defects in aprataxin cause the autosomal recessive neurodegenerative disorder Ataxia Oculomotor Apraxia 1 (AOA1) (8,9), which accounts for ∼10% of autosomal recessive cerebellar ataxias (10).

Despite substantial evidence pointing at distinct cellular roles for APTX, the DNA repair function and the consequence of APTX loss in cells and organs remain unclear (5,11–13). Studies on cultured vertebrate cells reveal minimal impact of APTX loss on cellular DNA repair capacity. The extent of this impact varies depending on the experimental read-out and the type of cells employed (5,6,13–15). *Aptx*−/− mice have been generated but they lack any obvious phenotype (1). This likely reflects the low frequency of APTX-dependent lesions and suggests that neuronal cell death occurs due to a stochastic accumulation of these lesions over time. Since the brain is protected from exogenous sources of damage by the cranium and the blood brain barrier, DNA lesions in the brain result predominantly from endogenous sources. The oxidative byproducts of cellular metabolic processes such as free radicals represent the main source of endogenous damage to DNA (16,17), which likely requires APTX's surveillance mechanisms to maintain DNA integrity. However, a suitable model system to study this putative role for APTX at the organismal level is not available.

To examine the role of APTX *in vivo*, we modulated the cellular antioxidant homeostasis by generating a mouse model where a mutant form of superoxide dismutase 1 (SOD1^G93A^) is expressed in an *Aptx*−/− background. SOD1^G93A^ mutation is associated with elevated levels of oxidative DNA breaks (18,19) and disrupted mitochondrial processes, causing motor neuron death observed in amyotrophic lateral sclerosis (ALS) (20). Here we report a defect in oxidative-induced DNA strand break repair and survival in *Aptx*−/− *SOD1^G93A^* double mutant cells above that observed for *Aptx*−/− or *SOD1^G93A^* single mutants. These observations translate well *in vivo* as shown by the reduced survival of spinal motor neurons in *Aptx*−/− *SOD1^G93A^* than *SOD1^G93A^* mice. Furthermore, the surviving neurons feature deep nuclear envelope invaginations, a hallmark of cellular stress. They also possess an elevated number of high-density nuclear regions with a concomitant increase in histone H3 K9 trimethylation, suggestive markers for silenced chromatin. The accelerated cellular senescence was also observed at the organismal level as shown by down-regulation of insulin-like growth factor 1 (IGF-1), a hallmark of premature ageing.

## RESULTS

**Figure 1.**
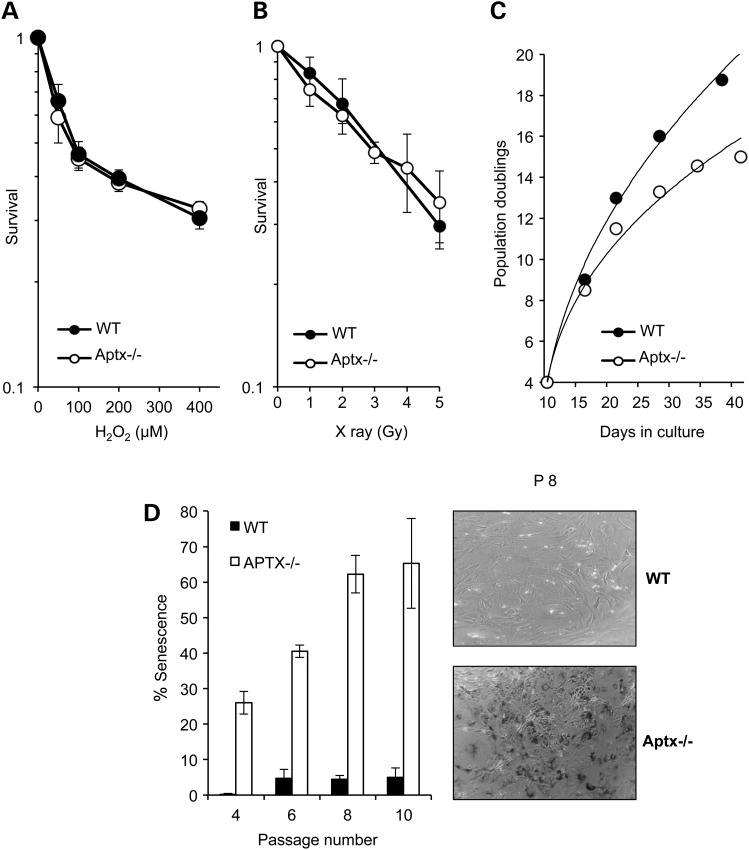
Accelerated cellular senescence in *Aptx*−/− cells*. Aptx+/+* ‘WT’ and *Aptx*−/− primary MEFs were exposed to the indicated doses of H_2_O_2_ (**A**) or X-rays (**B**) and percent survival quantified using clonogenic survival assays. Data are the average ± SEM from three biological replicates (**C**) Population doublings were calculated over 40 days of continuous culture and representative data are shown from one experiment. (**D**) MEFs were examined for senescence-associated β-gal (SA-βGal) staining at the indicated passages and β-Gal-positive cells were quantified and expressed as percent of total cells. The difference between WT and *Aptx*−/− MEFs was statistically significant at all passages examined (*P* < 0.01, two-way ANOVA) (*left*)*.* A representative image is depicted (*right*).

To examine the role of Aptx in response to oxidative stress, we first compared *Aptx*−/− and control mouse embryonic fibroblasts (MEFs) for their ability to survive H_2_O_2_-induced DNA damage (Fig. 1A). H_2_O_2_-induced a dose-dependent toxicity in control and *Aptx*−/− cells with no detectable difference in the rate or magnitude of cell death. Similarly, survival assays following exposure to ionizing radiation showed no significant survival defect in *Aptx*−/− MEFs (Fig. 1B). However, monitoring cellular growth over a period of 50 days showed a reduction in population doubling for *Aptx*−/− MEFs compared with their wild-type counterparts (Fig. 1C). We reasoned that the difference in growth rate might reflect the reduced ability of *Aptx*−/− MEFs to repair passage-associated accumulation of endogenous DNA damage. To test this, we compared control and *Aptx*−/− cells for levels of senescence-associated β-gal (SA-βGal), a widely used hallmark of senescence (21,22). Whilse wild-type cells exhibited <10% senescence over a period of 10 passages, *Aptx*−/− MEFs showed ∼25% senescence at passage 4 with a striking increase to 65% at passage 10 (Fig. 1D). We conclude from these experiments that *Aptx*−/− MEFs exhibit a passage-dependent increase of senescence compared with their wild-type counterparts.

**Figure 2.**
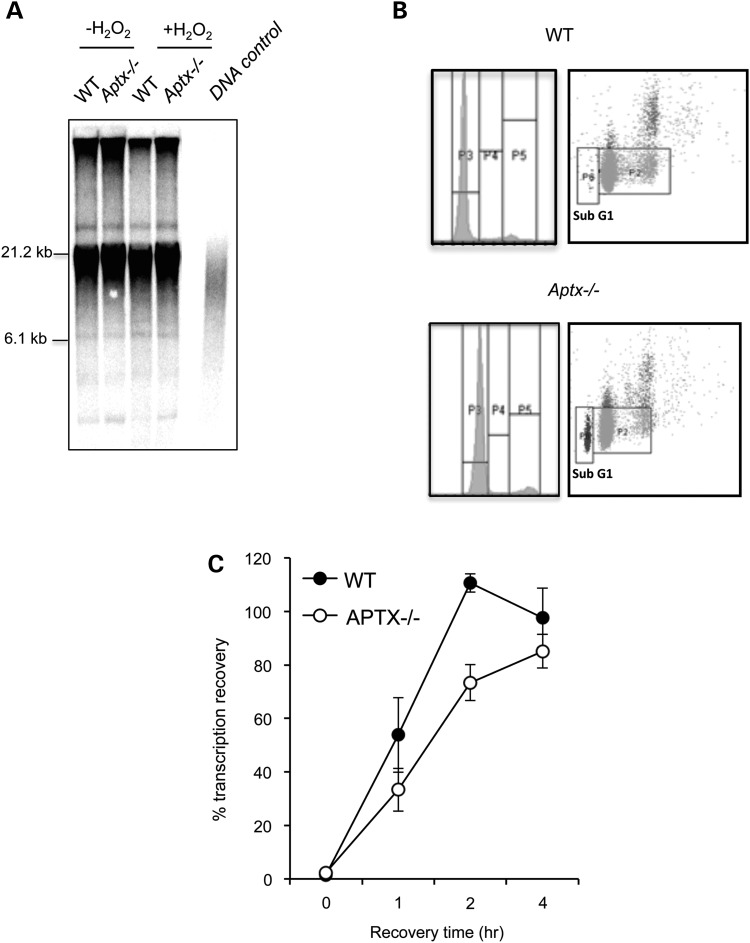
*Aptx*−/− cells show no detectable defect in telomere length but reduced transcription recovery following oxidative stress. (**A**) WT and *Aptx*−/− MEFs (passage 8) were mock incubated or incubated with pulsed treatment of 50 μm H_2_O_2_ for 10 min over three consecutive days. Genomic DNA was extracted and non-telemetric DNA was enzymatically digested and resolved by gel electrophoresis. Southern blotting was performed using a digoxigenin (DIG)-labelled probe specific for telomeric repeats (Roche) and incubated with a DIG antibody coupled to alkaline phosphatase. Telomeric signal was detected by chemiluminescence. Telomeric DNA control was obtained from Roche and used as size markers (not shown). (**B**) MEFs were fixed with cold ethanol, stained with propidium iodide and subjected to FACS analysis. A representative FACS profile is depicted. (**C**) The indicated MEFs were plated onto 3.5 cm dishes at 2 × 10^4^ cells per ml in medium containing 0.02 µCi/ml thymidine [2-^14^C] and incubated at 37°C for 48 h (∼2 cycles) to label DNA. Thymidine [2-^14^C] was removed and cells chased for 4 h and then mock treated or treated with 50 µM H_2_O_2_ for 10 min at room temperature. RNA synthesis was measured by incubation with 5 µCi/ml ^3^H-uridine for 15 min immediately after treatment or following subsequent incubation in H_2_O_2_-free media for the indicated recovery periods. RNA was quantified using Beckman Coulter LS6500 Scintillation Counter. RNA synthesis (^3^H count) was normalized to DNA content (^14^C count) and data plotted as % RNA synthesis relative to control un-treated samples from three biological replicates ± SEM.

Cellular senescence is the loss of replicative capacity in primary cells that can be triggered by accumulation of oxidative DNA breaks in telemetric regions of DNA, resulting in telomere uncapping and shortening (23). To test whether loss of Aptx affects telomere stability, we compared telomere length in control and *Aptx*−/− MEFs using the TeloTAGGG Telomere Length Assay. There was no detectable difference in telomere length between control and *Aptx*−/− cells (Fig. 2A) and repeated exposure to H_2_O_2_ for three consecutive days did not uncover a difference in telomere length either. These results suggest that the accelerated senescence in *Aptx*−/− cells was not due to a measurable telomere instability. Accelerated senescence was also unlikely to be caused by differences in cell cycle progression since fluorescence-activated cell sorting (FACS) analyses showed similar S/G2 distribution in wild-type and *Aptx*−/− MEFs (Fig. 2B). *Aptx*−/− MEFs, however, had a lower proportion of cells in G1 and a higher proportion of sub-G1 (dead) cells than controls, which is consistent with their accelerated senescence phenotype.

Next, we considered the possibility that the progressive accumulation of oxidative DNA breaks in *Aptx*−/− MEFs may interfere with progression of RNA polymerases (24,25), thereby accounting for the accelerated senescence and implicating Aptx in facilitating transcription recovery following oxidative damage. While incubation of cells with H_2_O_2_ resulted in a comparable reduction of total RNA levels in wild-type and *Aptx*−/− MEFs, subsequent incubation in H_2_O_2_-free media for 1 or 2 h revealed a significant (*P* < 0.01, *t*-test) delay in RNA synthesis in *Aptx*−/− MEFs (Fig. 2C). These experiments suggest that *Aptx*−/− MEFs are less able than controls to resume transcription following oxidative DNA damage.

**Figure 3.**
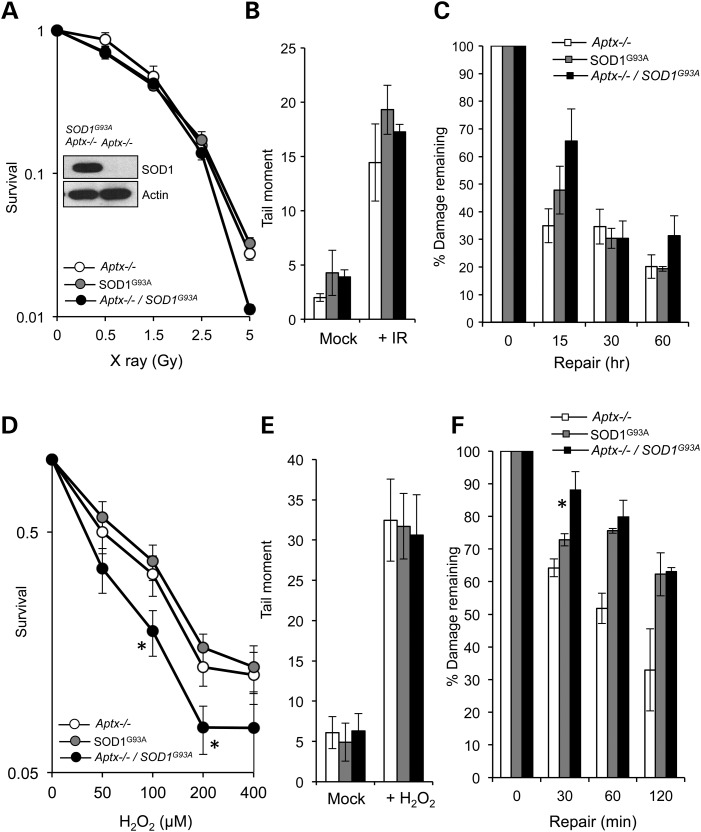
Expression of SOD1^G93A^ sensitizes *Aptx*−/− cells to oxidative stress. (**A**) *Aptx*−/−, *SOD1^G93A^*, *Aptx*−/− *SOD1^G93A^* MEFs were exposed to the indicated doses of X-ray and % survival quantified using clonogenic survival assays. Note that we did not include wild-type cells in these analyses since initial experiments showed similar and indistinguishable survival curves to *Aptx*−/− MEFs (Fig. 1). (**B** and **C**) MEFs were exposed to 20 Gy γ-irradiation and initial DNA strand breaks were quantified immediately after irradiation by alkaline comet assays. (**C**) Irradiated cells were incubated for the indicated repair periods in complete media and % DNA strand breakage remaining was quantified from three biological repeats. Data are the average ± SEM. (**D**) Cells were mock treated or treated with the indicated doses of H_2_O_2_ and % survival quantified from three biological replicates. Asterisks denote statistically significant difference between *SOD1^G93A^* and *Aptx*−/− *SOD1^G93A^* MEFs (*P* < 0.05, *t-*test). (**E**) Cells were treated with 90 μm H_2_O_2_ and DNA strand breakage quantified immediately after treatment using alkaline comets. (**F**) % DNA strand breaks remaining during subsequent incubation in H_2_O_2_-free media was quantified from three biological repeats. Asterisk denotes statistically significant difference between *SOD1^G93A^* and *Aptx*−/− *SOD1^G93A^* MEFs (*P* < 0.001).

Together, these observations show that *Aptx*−/− MEFs exhibit no detectable defect in global repair of oxidative DNA breakage yet they possess an accelerated senescence and reduced transcription recovery following acute doses of oxidative stress. Notably, *Aptx*−/− mice also exhibit no detectable phenotype, suggesting a lower cellular stress *in vivo* compared with culture conditions or perhaps a high level of physiological redundancy in response to Aptx deficiency. We reasoned that increasing the level of endogenous oxidative load might increase the cellular demand for Aptx and would enable us to uncover physiological functions that may not be measurable using canonical *Aptx*−/− models. We thus generated a mouse model in which *Aptx* loss is coupled with expression of the pathogenic SOD1^G93A^ protein. SOD1^G93A^ is a causative mutation for a familial form of amyotrophic lateral sclerosis (ALS), and is known to induce mitochondrial dysfunction and elevated oxidative stress (18,26–28). *Aptx*−/− *SOD1^G93A^* double mutant mice developed normally and their numbers followed the expected Mendelian ratios, suggesting little requirement for Aptx during embryonic development even in the presence of SOD1^G93A^. We next generated wild-type, *Aptx*−/−*, SOD1^G93A^* and *Aptx*−/− *SOD1^G93A^* double mutant MEFs and confirmed SOD1^G93A^ expression by immunoblotting (Fig. 3A, inset). Since the survival of wild-type MEFs were indistinguishable from *Aptx*−/− MEFs (Fig. 1A and B), we limited subsequent analyses to the latter. We first examined the effect of ionizing radiation (IR), which produces both direct DNA breaks and oxidative DNA damage via the hydrolysis of H_2_O. Exposure of cells to IR induced dose-dependent cell death in *Aptx*−/−*, SOD1^G93A^* and *Aptx*−/− SOD1^G93A^ cells with no significant difference in the rate or magnitude of cell death, at most doses examined (Fig. 3A). While IR induced comparable levels of DNA strand breakage in the three cell types (Fig. 3B), *Aptx*−/− *SOD1^G93A^* cells retained ∼70% of their breaks during a subsequent 15 min repair period, compared with ∼35% and 50% in *Aptx*−/− or *SOD1^G93A^* single mutants, respectively (Fig. 3C).

Since only a fraction of IR-induced damage is mediated by hydrolysis of H_2_O and generation of oxidative DNA damage, we next examined the effect of H_2_O_2_ which predominantly induces oxidative DNA breaks. Consistent with a role for Aptx during oxidative DNA break repair, *Aptx*−/− *SOD1^G93A^* double mutant MEFs were more sensitive to H_2_O_2_ than *Aptx*−/− or *SOD1^G93A^* single mutants (Fig. 3D). *Aptx*−/− *SOD1^G93A^* double mutants showed a significant survival defect at 100 and 200 μm H_2_O_2_ (*P* = 0.015 and *P* = 0.02, respectively) compared with Aptx-proficient *SOD1^G93A^* cells. In addition, despite starting with a similar number of breaks, *Aptx*−/− *SOD1^G93A^* double mutant cells retained more oxidative DNA breaks during 30 min repair periods than single mutants (Fig. 3E and F). These observations are unlikely to be due to differences in senescence rates between *Aptx*−/− *SOD1^G93A^* double mutants and *SOD1^G93A^* single mutants since they both exhibited comparable senescence (Supplementary Material, Fig. S1). Together, these data show that the expression of *SOD1^G93A^* sensitizes *Aptx*−/− cells to oxidative DNA damage.

**Figure 4.**
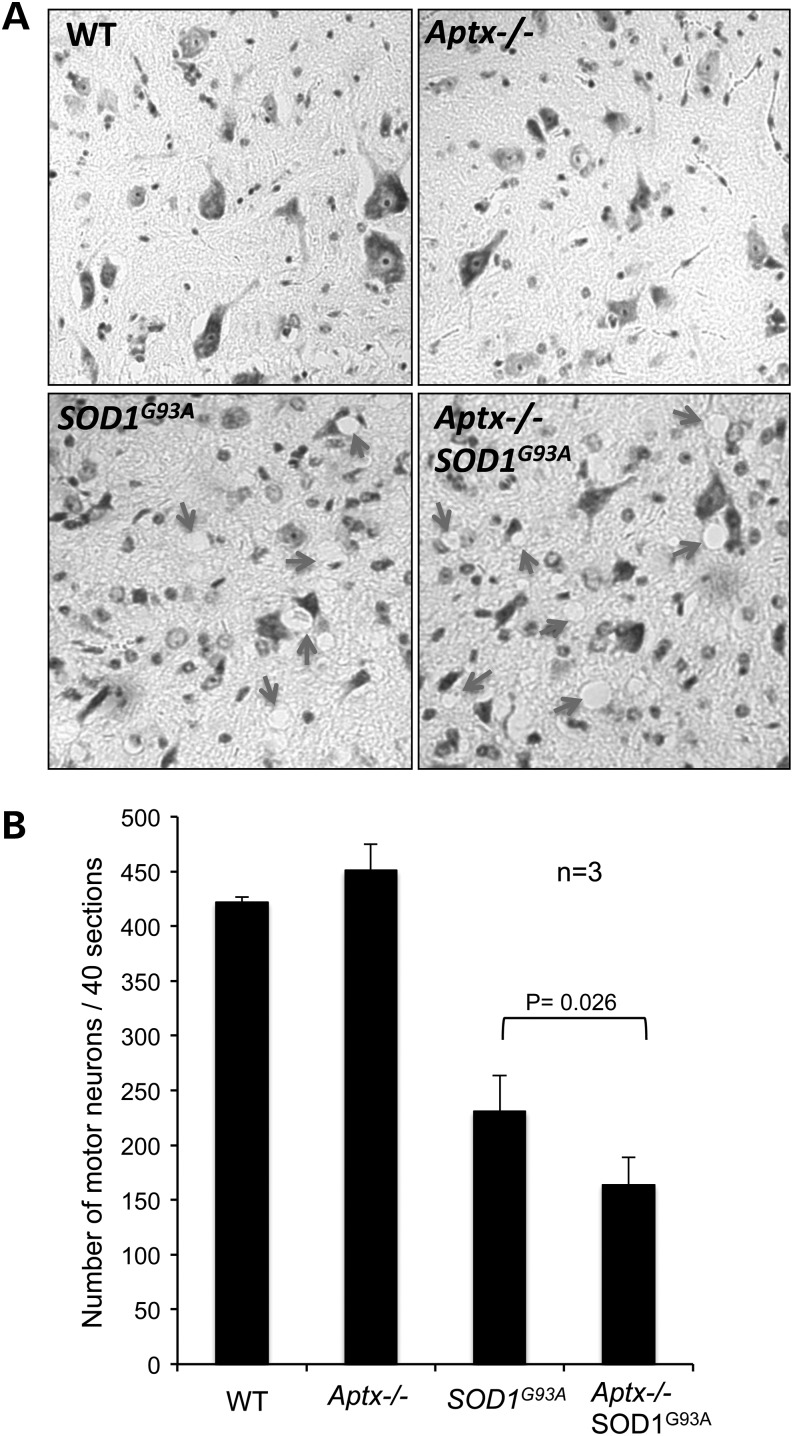
Reduced motor neuron survival in *Aptx*−/− *SOD1^G93A^* mice. Spinal cords were harvested from ∼3-month-old wild-type (WT) *Aptx*−/−, *SOD1^G93A^ Aptx*−/− *SOD1^G93A^* mice and lumbar regions were preserved in 3% neutral buffered formalin, embedded in paraffin blocks, and 10 μm sections were subjected to Nissl staining to quantify the number of motor neurons. A representative image is shown in (**A**). (**B**) The number of motor neurons from 40 sections was quantified from three independent littermates and data represent the average ± SEM. Statistical significance between *SOD1^G93A^* and *Aptx*−/− *SOD1^G93A^* motor neuron counts (*P* = 0.026) was calculated using student *t*-test.

Next we examined the consequences of SOD1^G93A^ overexpression in *Aptx*−/− cells/tissues *in vivo*. Expression of SOD1^G93A^ in mice causes neurological dysfunction, which is primarily manifested by hind limb paralysis and motor neuron defect (20,29,30). To test whether Aptx deficiency exacerbates the motor neuron loss in SOD1^G93A^ mice, we compared Nissl-stained motor neurons in spinal cord sections derived from wild-type, *Aptx*−/−, *SOD1^G93A^* and *Aptx*−/− *SOD1^G93A^* mice (Fig. 4). Wild-type and *Aptx*−/− motor neurons appeared healthy and intact whereas *SOD1^G93A^* and *Aptx*−/− *SOD1^G93A^* neurons were smaller and exhibited aberrant morphology (Fig. 4A). We observed a number of vacuoles in *SOD1^G93A^* and *Aptx*−/−*/SOD1^G93A^*, which were absent in wild-type and *Aptx*−/− tissue, suggesting positions of degenerated or degenerating neurons (arrow heads, Fig. 4A). This was not an artefact of staining since it was consistent in three independent experiments from three different litters. Subsequent quantification of the number of motor neurons revealed a remarkable reduction in motor neuron survival in *SOD1^G93A^* sections compared with *Aptx*−/− or wild-type controls (230 ± 30 SEM versus 450 ± 24 SEM; Fig. 4B). These observations are consistent with previous reports showing apoptotic cell death in *SOD1^G93A^* motor neurons (20,31). Importantly, deletion of *Aptx* in *SOD1^G93A^* mice resulted in significant additional reduction in motor neuron survival (230 ± 30 SEM versus 164 ± 25 SEM; *P* = 0.026; Fig. 4B).

**Figure 5.**
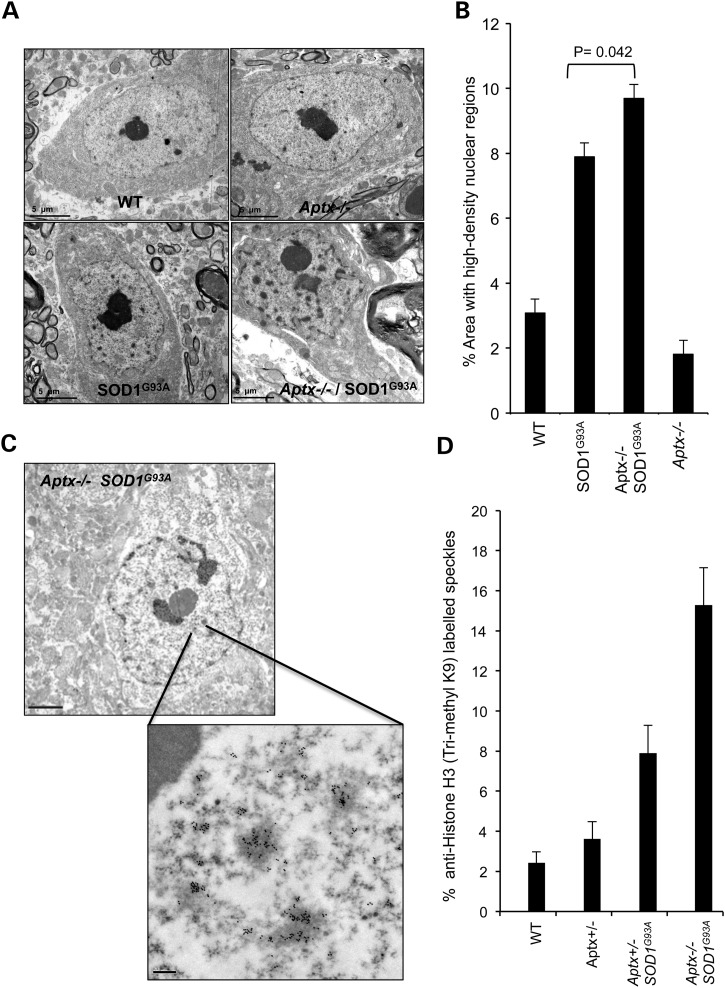
Increased nuclear invaginations and elevated Histone H3 K9 trimethylation in *Aptx*−/− SOD1^G93A^ neurons. (**A**) Representative transmission electron microscopy (TEM) images of motor neurons prepared for ultrastructural analysis from 3-month-old WT, *Aptx*−/−*, SOD1^G93A^* and *Aptx*−/− *SOD1^G93A^* mice. Scale bar = 5 μm. (**b**) The average nuclear area occupied by high-density nuclear regions was quantified from ∼70 neurons obtained from three independent litters. Data are the average ± SEM. (**C**) Representative TEM images of H3 K9 trimethylation revealed by immunogold labelling of motor neurons from 3-month-old *Aptx*−/− SOD1^G93A^ mouse. Scale bar = 2 μm (*top*) and 0.2 μm (*bottom*). (**D**) Average nuclear area occupied by the anti-Histone H3 (trimethyl K9)-positive dense chromatin within the indicated genetic backgrounds was quantified from 30 neurons obtained from three independent litters. Data are the average ± SEM.

To gain further insight into the consequences of *Aptx* deletion *in vivo,* we next examined the surviving fraction of motor neurons by transmission electron microscopy (TEM). Visual examination of TEM images revealed healthy nuclear envelopes in wild-type and *Aptx*−/− cells while abnormal envelops featuring numerous and deep invaginations were observed in *SOD1^G93A^* and *Aptx*−/− *SOD1^G93A^* neurons (Fig. 5A). These observations are intriguing since nuclear invaginations are characteristics of cellular stress that precede cell death, which is consistent with the lower number of neurons observed by Nissl staining (Fig. 4). Furthermore, we observed a significant increase in the number of dense chromatin in *SOD1^G93A^* and *Aptx*−/− *SOD1^G93A^* nuclei. While <3% of nuclear area was occupied by dense chromatin in wild-type and *Aptx*−/− neurons, >8% was observed in *SOD1^G93A^* and *Aptx*−/− *SOD1^G93A^* nuclei (Fig. 5B). Notably, *Aptx*−/− *SOD1^G93A^* nuclei had ∼2% increase of dense chromatin compared with *SOD1^G93A^* nuclei.

The high-density regions in the nucleus are likely nuclear speckles, representing regions of chromatin condensation. Speckles or interchromatin granule clusters are dynamic structures that could be involved in the coordination of transcription and pre-mRNA splicing. It is also likely that the majority of the high-density regions observed here represent areas occupied by condensed or silenced chromatin, which presumably arise due to progressive accumulation of unrepaired DNA breaks in *Aptx*−/− *SOD1^G93A^* neurons. To ascertain the nature of the observed dense chromatin, we carried out immunogold labelling TEM with anti-histone H3 trimethyl lysine 9 (H3K9me3) antibody, a hallmark for heterochromatin. Consistent with these high-density regions being sites of silenced chromatin, ∼14% of *Aptx*−/− *SOD1^G93A^* nuclear area was enriched with H3K9me3 compared with ∼8% and ∼3% for *SOD1^G93A^* and controls, respectively (Fig. 5C and D). We conclude from these experiments that expression of a toxic form of SOD1 increases the proportion of heterochromatin in *Aptx*−/− neurons.

**Figure 6.**
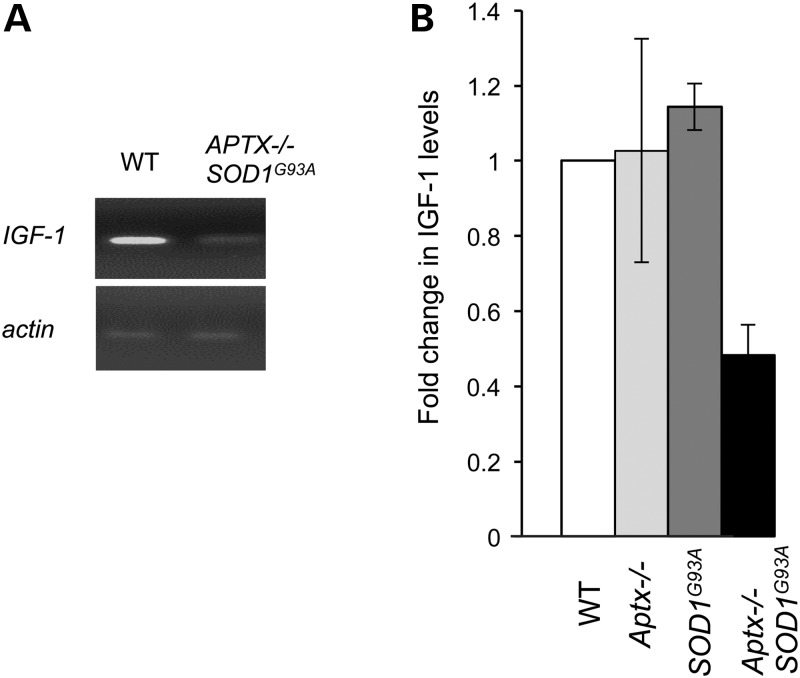
*Aptx*−/− *SOD1^G93A^* mice feature molecular hallmarks of organismal ageing. Electrophoretic analysis of the products of RT-PCR amplification of β-actin (control) and insulin-like growth factor (*IGF-1)* from mRNA extracted from liver of 3-month-old WT, *Aptx*−/−, *SOD1^G93A^* and *Aptx*−/− *SOD1^G93A^* mice. A representative image is depicted in (**A**) and quantification of the fold change in *IGF*-1 levels (by qPCR) relative to the level observed in WT mice is shown in (**B**). Data are the average of three independent replicates ± SEM.

Finally, the accelerated senescence (Fig. 1), the reduced ability to repair oxidative DNA breaks (Fig. 3) and the elevation of silenced chromatin (Fig. 5) are consistent with signs of premature ageing at the cellular level. To examine whether this is also true at the organismal level we examined the expression of insulin-like growth factor 1 (*IGF-1*), a molecular hallmark of premature ageing. Circulating growth hormone is converted into IGF-1 in the liver and persistence of transcription-blocking DNA breaks has been shown to down-regulate IGF-1 in nucleotide excision repair defective progeria rodent models (32). Reverse transcriptase-polymerase chain reaction (RT-PCR) analysis of β-actin (control) and *IGF-1* mRNA transcripts extracted from liver of 3-month-old wild-type, *Aptx*−/−, *SOD1^G93A^* and *Aptx*−/− *SOD1^G93A^* mice revealed a reduction of *IGF-1* expression in the latter (Fig. 6A). Subsequent quantitative PCR analyses of cDNA generated from adult mouse livers revealed ∼3-fold reduction (*P* < 0.001) of *IGF-1* expression in *Aptx*−/− *SOD1^G93A^* compared with other genotypes tested (Fig. 6B). Together, these data demonstrate a protective role for Aptx at the cellular and organismal level, and suggest that its absence results in progressive accumulation of oxidative DNA breaks in the nervous system, triggering hallmarks of premature ageing, systemically.

## DISCUSSION

APTX deficiency causes progressive cerebellar degeneration, ataxia and oculomotor apraxia in man (8,9). Multiple *in vitro* studies have shown that APTX cleaves AMP from 5′-DNA termini during premature ligation cycles (1,15,33–35). Incomplete ligation cycles can take place due to the unavailability of 3′-hydroxyl groups, which are normally lost in most, if not all, DNA break events (2–4). Studying the physiological significance of APTX has been hindered by the lack of the appropriate model systems. For example, *Aptx* deletion in mice results in no overt phenotype, which likely reflects the low frequency of 5′-AMP DNA breaks and suggests that neuronal cell death occurs due to stochastic accumulation of DNA breaks over time (1,9,36–39). To overcome these limitations, we generated a murine model system where a pathogenic mutant of superoxide dismutase 1 (SOD1^G93A^) is expressed in an *Aptx*−/− mouse strain. We analyzed the single and double mutant cells and tissues for their growth rate, survival, chromosomal break repair proficiency, nuclear architecture and hallmarks of premature aging.

We did not detect a survival defect following oxidative DNA damage in *Aptx*−/− primary murine fibroblasts, which is consistent with results obtained from murine astrocytes (3) and human AOA1 cells (5). However, monitoring cellular growth over a period of ∼2 months uncovered a reduction in population doubling in *Aptx*−/− MEFs compared with controls. We suggest that this difference in growth rate is due to a progressive accumulation of passage-associated endogenous DNA damage at low oxygen culture conditions (3% O_2_). In support of this prediction *Aptx*−/− MEFs, but not control cells, possess a progressive increase of senescence rate, starting from ∼25% senescence at passage 4 to ∼65% at passage 10. The delayed growth and elevated senescence rates were not due to detectable telomere instability or differences in cell cycle progression, as shown by telomere assays and FACS profiling. Transcription recovery assays on the other hand suggest a limited ability of *Aptx*−/− cells to recover transcription following fractionated doses of H_2_O_2_. Un-repaired 5′-AMP DNA breaks could interfere with cellular transcription and may affect global gene expression, accounting for the delayed growth and elevated senescence rates observed in this study. This is consistent with studies from the Lavin laboratory that showed reduced expression of several DNA repair enzymes such as PARP-1, apurinic endonuclease 1 (APE1) and OGG1 in AOA1 cells (13). This is also consistent with reports showing association of APTX with nucleolar proteins such as nucleolin and nucleophosmin (5,6). The transcription defect may also explain the unique extraneurological features of AOA1 such as decreased serum albumin (9). Notably, albumin is an abundant protein with antioxidant properties and as such any reduction in its steady state level would cause further sensitization to oxidative damage (40).

Modulating cellular oxidative homeostasis by overexpression of a pathogenic mutant of SOD1 uncovered a survival and chromosomal break repair defect in *Aptx*−/− cells. It is notable that overexpression of SOD1^G93A^ in *Aptx*−/− cells did not result in a detectable increase in H_2_O_2_-induced DNA breaks but led to significant delay in repair rates during a short 30 min repair periods. This likely reflects a SOD1^G93A^-mediated increase in the frequency of premature ligation events during repair. This may take place during the unsuccessful attempts of DNA ligases to seal DNA breaks during incubations in H_2_O_2_-free media despite being produced at equal frequency. Overexpression of SOD1^G93A^ may elevate the frequency of premature ligations during single-strand break repair (36) or during the process of ribonucleotide clearance from genomic DNA (2). The latter is intriguing due to the greater cellular concentration of ribonucleotides, which favours their ‘erroneous’ incorporation in genomic DNA at high rates, with estimates exceeding 100 000 ribonucleotides per cell division in vertebrate cells (41). RNase H2 initiates the incision of ribonucleotides from DNA, leaving behind a 5′-ribose residue. The failed attempt of DNA ligases to seal this intermediate results in adenylated 5′-ribose termini, channelling repair to an APTX mediated process to remove the AMP and reset the ligase (2). It is likely that overexpression of SOD1^G93A^ increases the demand for the RNase H2-dependent removal of ribonucleotides from DNA, thereby increasing the abundance of premature ligation events. The increased reliance on RNase H2 incisions may be caused by SOD1^G93A^-mediated trapping of redundant enzymes involved in ribonucleotide incision and removal such as topoisomerase 1 (Top1) (42). Consistent with this possibility, several studies have shown that oxidative breaks, nicks and gaps can trap Top1 on DNA (24). Such breaks would not be detectable by the comet assay hence no difference was observed immediately following H_2_O_2_ treatment (17). Although our data do not provide a conclusive explanation for the observed cellular defect it provides a pool of testable hypotheses and resources for further investigation.

The chromosomal break repair defect in SOD1^G93A^ *Aptx*−/− cells reflects a role for APTX in nuclear DNA repair since the comet assay does not detect mitochondrial DNA breaks (15,43). However, the survival defect could reflect additional roles for APTX in the mitochondria (7,44). In support of this possibility it is worth noting that the survival defect observed *ex vivo* in cultured cells was also observed in tissues. Spinal cord sections from *Aptx*−/− *SOD1^G93A^* mice showed a reduction in motor neuron survival compared with sections from *SOD1^G93A^* mice. Analyses of the surviving fraction of motor neurons by transmission electron microscopy (TEM) revealed unhealthy nuclear envelops with deep invaginations and an elevated number of high-density nuclear regions. These observations are hallmarks of cellular stress that precede cell death and are consistent with the survival defect and the accelerated senescence rate observed *ex vivo*. There are several explanations for the nature of the high-density nuclear regions observed in this study: (i) they could represent dynamic structures demarking sites of nuclear speckles, which are involved in the coordination of transcription, pre-mRNA splicing and mRNA export (45,46), (ii) they could represent sites of sequestration of factors that regulate expression of genes involved in the stress response known as nuclear stress bodies (47), or (iii) they could represent areas of dense or silenced chromatin. Immunogold labelling TEM analyses using anti-histone H3K9me3 antibody, a hallmark for heterochromatin, favours the third possibility and suggests that the high-density regions are silenced chromatin, likely due to the progressive accumulation of unrepaired DNA breaks in *Aptx*−/− *SOD1^G93A^* tissues.

Together these findings are consistent with a model whereby progressive accumulation of DNA damage in *Aptx*−/− *SOD1^G93A^* tissues exacerbates cell death *in vivo* and senescence *ex vivo*. The latter has also been observed *in vivo* as shown by the down-regulation of IGF-1, a hallmark of premature ageing (32). IGF-1 is primarily produced in the liver as an endocrine hormone and in target tissues as a paracrine/autocrine factor. Its production is stimulated by growth hormone and can be inhibited by attenuation of the growth hormone response pathway. The data presented here are consistent with the proposal that progressive accumulation of transcription-blocking DNA lesions down-regulates the somatotropic axis and reduces the expression of IGF-1. This is supported by several reports showing that persistent transcription-blocking lesions trigger somatic growth attenuation in order to switch physiological status from somatic ‘growth’ to ‘maintenance’ in an attempt to tolerate persistent transcription-blocking DNA lesions (32,48). In agreement with this, the double mutant *Aptx*−/− *SOD1^G93A^* mice showed a subtle reduction in body weight compared with single *SOD1^G93A^* mice (Supplementary Material, Fig. S2). The limited life span of *Aptx*−/− *SOD1^G93A^* mice prevented us from assessing other hallmarks of premature ageing such as kyphosis and grey hair. Further studies will employ another variant of SOD1^G93A^ mice that carries a deletion of the transgene array to reduce the copy number and thus undergoes a slower course of disease (49). We anticipate this approach would give us sufficient window to observe hallmarks of ageing at the organismal level. The observed transcription recovery defect, down-regulation of IGF-1 and early senescence are consistent with somatic growth attenuation in stressed cells lacking APTX and indeed, this seems to be a logical coping strategy which may not be too surprising. Under situations of cellular stress, somatic attenuation can lead to a range of compensatory effects including suppression of the production of reactive oxygen species and promotion of DNA repair. In the case of loss of APTX, this somatotropic attenuation may be sufficient to avoid impact on cycling cells and protect neurons in lower eukaryotes sufficiently long enough for them to never develop symptoms. However, in humans, who have especially high cerebellar activity coupled with long lifetimes, it is likely insufficient to rescue neural viability and the most sensitive cells eventually cease to function sufficiently and/or die, resulting in AOA1 pathology.

It is interesting to note that there was a significant transcription recovery defect in *Aptx*−/− cultured cells relative to wild-type but no sizeable down-regulation in IGF-1 expression in the livers of genetically similar aged animals, whereas *Aptx*−/− *SOD1^G93A^* exhibited a significant down-regulation in IGF-1 expression in the liver. This suggests that the down-regulation of IGF-1 may result from an active process in response to exceeding a pre-set threshold of DNA damage and perhaps specific to certain regions of the genome. The effect may also be tissue specific, for example, a threshold level of DNA damage in the pituitary (which secretes the growth hormone required for the production of IGF-1 in the liver) or the hypothalamus (which regulates growth hormone release) may trigger a signal to reduce IGF-1 production in the liver. Alternatively, persisting damage in the liver may prevent IGF-1 formation. Since the nervous system is especially sensitive to DNA damage and IGF-1 is a circulating hormone, it is tempting to speculate that the effect is a systemic one driven by DNA damage in the nervous system. While the present data do not pin down the exact mechanism of IGF-1 down-regulation, they provide a model system and a testable hypothesis. Further research is required to trace the origins of IGF-1 down-regulation by measuring growth hormone levels and downstream signalling components and by comparing expression of relevant components of the signalling cascade in various tissues.

## MATERIALS AND METHODS

### Generation of *Aptx*−/− and *Aptx*−/− *SOD1^G93A^* mice and mouse embryonic fibroblasts

*Aptx*−/− mice were generated as described previously (1,3). *Aptx*+/− mice were mated with *SOD1^G93A^* mice (20) to generate *Aptx*+/− *SOD1^G93A^*, which were backcrossed with *Aptx*+/− to generate *Aptx*+/+, *SOD1^G93A^*, *Aptx*−/− and *Aptx*−/− *SOD1^G93A^* mice. For the generation of mouse embryonic fibroblasts (MEFs), embryos were harvested at Embryonic day 13, heads and red organs were separated and the body homogenized in trypsin, and then cultured in complete medium [Dulbecco's modified Eagle's medium (DMEM) 15% FCS at 37°C, 3% O_2_, 5% CO_2,_ 100 units/ml penicillin, 100 mg/ml streptomycin, 2 mm l-glutamate). Genotyping of adult mice was conducted using tail biopsies and genotyping of embryos was done using the embryo heads and was further confirmed by repeating the PCR on cultured MEF lysates. Cells/tissues were digested in lysis buffer (20 mm Tris–HCl pH 7.5, 10 mm EDTA, 1 mm EGTA, 100 mm NaCl, 1% Triton X-100 proteinase K (Invitrogen) at 10 mg/ml final concentration), followed by incubation at 55°C overnight. DNA was diluted 1:50 and used as a template in PCR reactions as described previously (1). The primers used were APTX-1: AGCAAGTGGCCTCACATACACATGC, APTX-2: CTCCTTGCCTGCTTACACTCCAGC and APTX-3: TTCTCTCCATGACTGGTCATGGC from Eurofins. Genotyping for *SOD1^G93A^* was performed using primers CATCAGCCCTAATCCATCTGA and CGCGACTAACAATCAAAGTGA (Eurofins). All animals were housed and maintained in accordance with the institutional animal care and ethical committee at the University of Sussex.

### Immunoblotting

To examine the expression of Aptx, endogenous murine SOD1 and ectopic human SOD1^G93A^, ∼5 × 10^5^ cells were harvested by centrifugation at 1500 rpm and washed three times in phosphate buffered saline (PBS). Cell pellets were lysed by incubation with 0.2 ml cell lysis buffer [20 mm Tris–HCl pH 7.5, 10 mm EDTA, 1 mm EGTA, 100 mm NaCl, 1% Triton X-100 and 1/100 dilution of protease inhibitor cocktail (Sigma)] on ice and cell free extracts were prepared by centrifugation at 13 000 rpm for 20 min at 4°C. The supernatant was retained and protein concentration quantified by the Lowry method using the *DC* Protein Assay (BioRad) with BSA used as a standard. Cell free extracts were fractionated using 12.5% sodium dodecyl sulphate-polyacrylamide gel electrophoresis. Proteins were transferred to a Hybond C extra nitrocellulose membrane at 25 V for 2 h. Following transfer, membranes were blocked by 5% milk/PBS for 1 h at room temperature, and incubated overnight at 4°C with the following primary antibodies: rabbit anti-aprataxin (1:1000, 5% milk, MW ∼40 kDa), rabbit anti-SOD1 (Santa Cruz Biotechnology, MW ∼18 kDa) in 5% milk. Membranes were washed in Tris-Buffered Saline/Tween 20 (50 mm Tris, 150 mm NaCl, 0.05% Tween, pH 7.6) and then incubated at room temperature for 1 h in goat anti-rabbit HRP-conjugated secondary antibody (Dako). Membranes were washed again and incubated in enhanced chemiluminescence (ECL) western blotting detection reagent (GE Healthcare) and developed by autoradiography using a Xograph Compact 4 automatic X-ray film processor.

### Cell growth and survival assays

Primary MEFs were employed at passage 4–6 for survival assays. Of note, 100–600 cells per ml were grown in 10 ml plates (triplicates) and treated with the appropriate DNA damaging agent: X-ray 250 kV at 12 mA, dose rate 0.5 Gy/min using the AGO 320/250 X-ray cabinet or H_2_O_2_ in PBS at the indicated doses for 10 min at room temperature. Cells treated with X-ray were immediately incubated at 37°C for 5–7 days, and cells treated with H_2_O_2_ were washed three times in PBS and then incubated at 37°C. In each experiment, cells were allowed to grow for the same period, between 5 and 7 days, until colonies had formed. Media were removed and cells fixed with 80% ethanol for 2 min, then ethanol was removed. One percent methylene blue stain was added to cover the bottom of the plate, and left for 30–40 min. Stain was removed and plates left upside down to dry before colonies were counted. The surviving fraction of cells of each genotype at each dose was calculated according to the number of colonies formed and plating efficiency, using the equations: plating efficiency = colonies observed/number of cells plated, Surviving fraction = colonies observed/cells seeded × plating efficiency. The average surviving fraction from at least three biological replicates were calculated ±SEM. To determine population doubling time, primary MEFs (P1) were plated at a density of 6 × 10^4^ cells/ml, grown to ∼80% confluency, counted and replated. This process was repeated over 40 days of continuous culture and a representative data are shown from one experiment.

### Senescence-associated β-gal (SA-βGal) assays

The senescence of primary MEFs was assayed at alternate passages up to passage 10, using the Senescence β-Galactosidase Staining Kit (Cell Signalling), which exploits pH-dependent β-Galactosidase activity as a readout for senescence. Primary MEFs were seeded at ∼6 × 10^4^ cells/ml, allowed to grow for ∼72 h, fixed with 80% ethanol for 10 min at each passage, then stained with X-Gal (20 mg/ml) and incubated at 37°C for 24–72 h. β-Gal-positive cells were quantified using 200× magnification using an optical microscope and expressed as percent of total cells.

### RNA synthesis recovery assays

MEFs (passage 4–6) were plated onto 3.5 cm dishes at ∼2 × 10^4^ cells in 15% FCS-medium containing 0.02 µCi/ml thymidine [2-^14^C] and incubated at 37°C for 48 h (∼2 cycles) to label DNA. Medium was removed, cells washed with PBS, and incubated with either PBS or 50 µM H_2_O_2_ for 10 min at room temperature. Cells were washed three times with PBS, and incubated in media containing 5 µCi/ml ^3^H-uridine for 15 min to label RNA. Cells were washed three times with PBS and 0.25 ml 2% SDS was applied and cells scraped off the dish with a silicone rubber to collect DNA and RNA. Strips of Grad 17 Whatman chromatography paper was marked into 16 × 1-in. labelled sections. Hundred microliter samples were pipetted onto paper strips, in duplicate. Strips were incubated with 5% TCA for 5 min followed by 2 × 5 min incubation with IMS. Finally, samples were left to dry thoroughly and radioactivity was quantified using the Beckman Coulter LS6500 Multi-Purpose Scintillation Counter. RNA synthesis (^3^H count) was normalized to DNA content (^14^C count) and data plotted as % RNA synthesis relative to control un-treated samples.

### Alkaline single-cell gel electrophoresis

2 × 10^5^ MEFs at passage 5 were plated onto 3.5 cm culture dishes in complete media and incubated for 10 h at 37°C prior to mock-treatment or treatment with 90 μm H_2_O_2_ on ice for 10 min. For exposure with γ-rays, 4 × 10^5^ cells/ml were irradiated in suspension in complete medium using a ^137^Cs gamma source (dose rate, 7.5 Gy/min). After treatment with H_2_O_2_ or γ-rays cells were washed in ice-cold PBS and incubated for the indicated repair periods. Cells were harvested in ice-cold PBS and analyzed by alkaline comet assays as described previously (43). DNA was neutralized, stained with SYBR green (1:10 000 dilution) and visualized by fluorescence microscopy (Nikon Eclipse E400) at 20× magnification. Comet tail moment for 100 cells per sample was determined using Comet Assay IV software (Perceptive Instruments).

### Telomere stability assays

Telomere assays were performed using the TeloTAGGG Telomere Length Assay kit from Roche. Briefly genomic DNA was extracted from MEFs, digested with the frequently cutting restriction enzymes *Hinf*1 and *Rsa*1. The sequence specificity of theses enzymes ensures that only non-telomeric DNA is digested to low molecular weight fragments. DNA fragments were separated by gel electrophoresis, transferred onto a nylon membrane and subjected to Southern blotting using digoxigenin (DIG)-labelled probe-specific for telomeric repeats. Membranes were incubated with a DIG-specific antibody covalently coupled to alkaline phosphatase and signals visualized by chemiluminescence.

### FACS analysis

1 × 10^5^–1 × 10^6^ MEFs at passage 4–6 were suspended in 200 μl cold PBS and 700 μl ice-cold ethanol was added dropwise while vortexing to avoid clumping. Cell suspension was left at −20°C for 2 h. Cells were washed twice with PBS and then incubated with 50 μg/ml propidium iodide containing 1× RnaseA (Sigma). Cells were gated according to their cell cycle distribution and analyzed using FACSCanto (BD Biosciences).

### IGF-1 expression

RNA was extracted from adult mouse liver (aged 3 month) using the RNeasy Plus Mini Kit (Qiagen), checked for DNA contamination using gel electrophoresis, and then cDNA was generated using the Promega reverse transcription kit. Briefly, 2 μg RNA and 0.5 μg oligo dT were incubated at 70°C for 5 min and then added to a master mix containing 10 mm dNTPs, recombinant Rnasin^®^ Ribonuclease Inhibitor (25 units), and M-MLV reverse transcriptase (200 units), and incubated at 42°C for 1 h. One microliter RNase A (Promega) was added and the mixture was incubated at 37°C for 30 min. cDNA was purified with the QIAquick Spin PCR purification kit and 2.5 μl of the product was employed in a qPCR reactions with Quantitect SYBR® Green PCR Master Mix (Qiagen) containing 100 nm each of the appropriate forward and reverse primers (β-actin forward: GACGTTGACATCCGTAAAGA, β-actin reverse: AATCTCCTTCTGCATCCTGT, IGF-1 forward: TGCTTGCTCACCTTCACCA, IGF-1 reverse: CAACACTCATCCACAATGCC) from Eurofins. Reaction products were analyzed using Stratagene MX4000 qPCR and average fold changes in expression was quantified from three independent biological replicates.

### Motor neuron count

The spinal cord was isolated from ∼3-month-old mice and lumbar regions were preserved in 3% neutral buffered formalin and embedded in paraffin. Serial 10-μm transverse sections were cut and stained with gallocyanin, a Nissl stain. The number of Nissl-stained motor neurons in the sciatic motor pool in a total of 40 sections per mouse, between the L3 and L6 levels of the spinal cord were counted. Only large, polygonal neurons with a distinguishable nucleus and nucleolus and a clearly identifiable Nissl structure were included in the counts.

### Transmission electron microscopy

Lumbar spinal cords were harvested from 3-month-old mice and fixed in 4% neutral buffered formalin for 72 h at 4°C. Subsequently, the samples were cut into two, with one-half processed for ultrastructural analysis and the other half for immunogold labelling transmission electron microscopy (TEM) as follows. For ultrastructural analysis, the samples were additionally fixed in 2.5% glutaraldehyde in 0.1 M NaPO_4_ buffer, pH 7.4, for 2 h at room temperature, then left overnight at 4°C. After buffer rinsing, samples were post-fixed in 1% osmium tetroxide in phosphate buffer, rinsed in distilled water, dehydrated in an ethanol series, passed through propylene oxide, and embedded in TAAB Low Viscosity resin (TAAB Laboratories Ltd., Aldermaston, UK). For immunogold labelling, the samples were prepared by a minimal, cold fixation and embedding in Unicryl resin (British BioCell International, Cardiff, UK) procedure, as we have previously described in detail (50,51). Thin sections (100 nm) of the fixed and embedded samples were cut on a Leica Ultracut ultramicrotome (Leica Microsystems, Milton Keynes, UK) and collected upon nickel TEM support grids. For immunogold labelling, these latter were immersed in a solution of sellotape dissolved in chloroform prior to use, to enhance section adherence during the labelling procedure. We used established methods to perform immunogold labelling TEM (52), which, briefly were as follows. A modified PBS solution, pH 8.2, consisting of 1% BSA, 500 µl/l Tween-20, 10 mm Na EDTA and 0.2 g/l NaN_3_ was used throughout the following procedures for all dilutions of antibodies and secondary gold probe. Thin sections were initially blocked in normal goat serum (1:10) for 30 min at room temperature, then incubated in a rabbit polyclonal anti-Histone H3 (trimethyl K9) antibody (Abcam plc [Ab8898], Cambridge, UK) at 10 μg/ml IgG final concentration overnight at 4°C. After 3 × 2 min PBS+ rinses, the sections were immunolabelled with 10 nm gold particle-conjugated goat anti-rabbit IgG secondary probe (1:10 for 1 h at room temperature; British BioCell International, Cardiff, UK). Sections were subsequently rinsed in PBS+ (3 × 10 min) and distilled water (4 × 5 min). All thin sections (for immunogold labelling or ultrastructural analysis) were subsequently post-stained in 2% (w/v) aqueous 0.22 μm-filtered uranyl acetate for 1 h. The thin sections were examined in a Hitachi 7100 TEM at 100 kV, systematically scanned for motor neurons and images acquired digitally with an axially mounted (2K × 2K) Gatan (Gatan UK, Abingdon, UK) Ultrascan 1000 CCD camera.

### Quantification of nuclear high-density regions

Image J computer software, developed by the National Institutes of Health (USA), was used to assess nuclear morphology. For the assessment of the percentage area of high-density regions within the nucleus (nuclear speckles), the areas of the nucleolus and nucleus were initially measured using a thresholding technique, pixels under a set level of darkness were removed from the image so that only pixels representing high-density nuclear regions were left. The total area of darker regions, excluding areas smaller than 250 pixels in size, were measured and this corresponded to the total high-density areas in the nucleoplasm. By calculating the fraction of total nuclear area, excluding the nucleolus, composed of darker pixels, the percentage area of high-density regions within the nucleus was obtained.

## SUPPLEMENTARY MATERIAL

Supplementary Material is available at *HMG* online.

## FUNDING

This work is funded by an Ataxia UK grant, a Wellcome Trust Investigator Award (103844), and a Lister Institute of Preventative Medicine Fellowship to S.E.-K. L.G. is the Graham Watts Senior Fellow, supported by the Brain Research Trust and the European Community's Seventh Framework Programme (FP7/2007-2013). Funding to pay the Open Access publication charges for this article was provided by the Wellcome Trust.

## Supplementary Material

Supplementary Data
