# Supplementary material for: Expression of a pathogenic mutation of SOD1 sensitizes aprataxin-deficient cells and mice to oxidative stress and triggers hallmarks of premature ageing
Source: Hum Mol Genet. 2014 Sep 30;24(3):828–40. doi: 10.1093/hmg/ddu500 (PMC4291253; doi:10.1093/hmg/ddu500)
Supplement: Supplementary Data [file supp_24_3_828__index.html]

Expression of a pathogenic mutation of SOD1 sensitizes aprataxin-deficient cells and mice to oxidative stress and triggers hallmarks of premature ageing — Expression of a pathogenic mutation of SOD1 sensitizes aprataxin-deficient cells and mice to oxidative stress and triggers hallmarks of premature ageing — Supplementary Data 

# Expression of a pathogenic mutation of SOD1 sensitizes aprataxin-deficient cells and mice to oxidative stress and triggers hallmarks of premature ageing

## Supplementary Data

Supplementary Data

**Files in this Data Supplement:**

- Supplementary Data - Pdf file
