# Supplementary material for: Expression of a pathogenic mutation of SOD1 sensitizes aprataxin-deficient cells and mice to oxidative stress and triggers hallmarks of premature ageing
Source: Hum Mol Genet. 2014 Sep 30;24(3):828–40. doi: 10.1093/hmg/ddu500 (PMC4291253; doi:10.1093/hmg/ddu500)
Supplement: Supplementary Data [file supp_ddu500_ddu500supp.pdf]

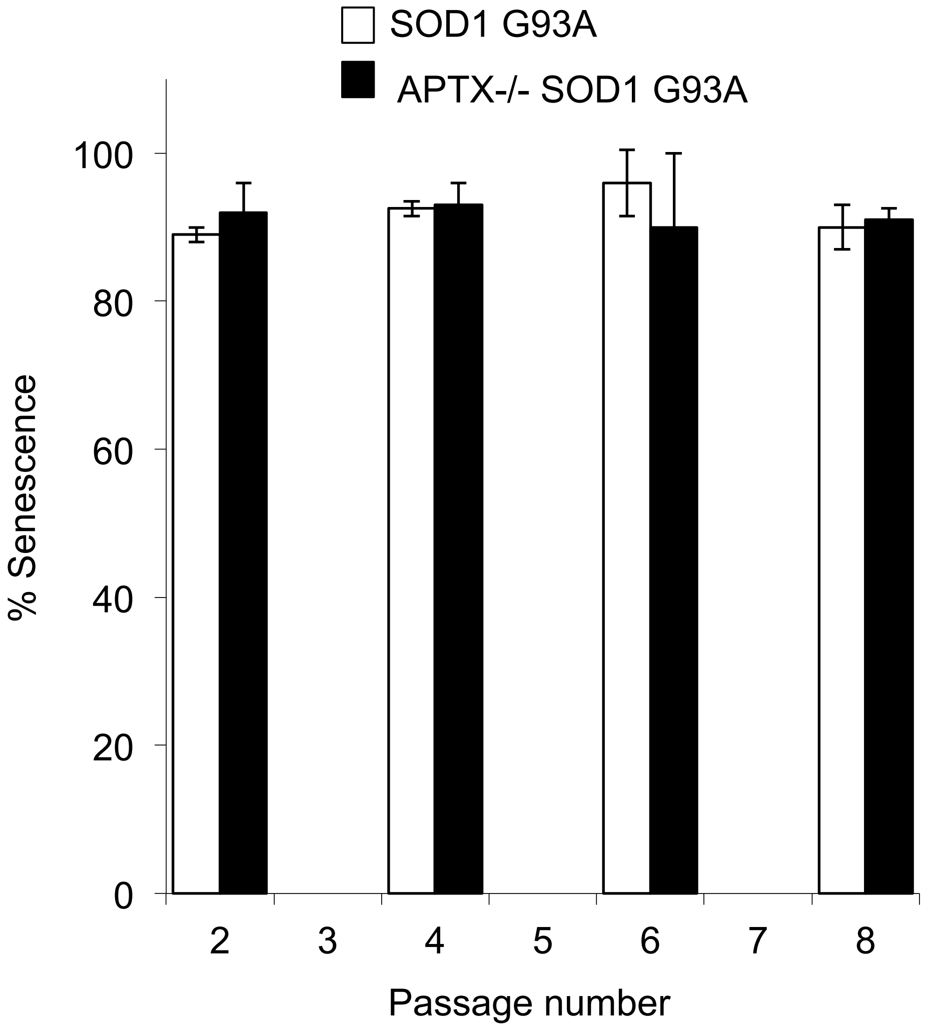

Supplementary figure 1. SOD1 G93A MEFs exhibit high senescence rate that is not affected by additional deletion of *Aptx. Primary MEFs were examined for senescence-associated β-gal (SA-βGal) staining at the indicated passages and β-Gal positive cells were quantified and expressed as % of total cells.*

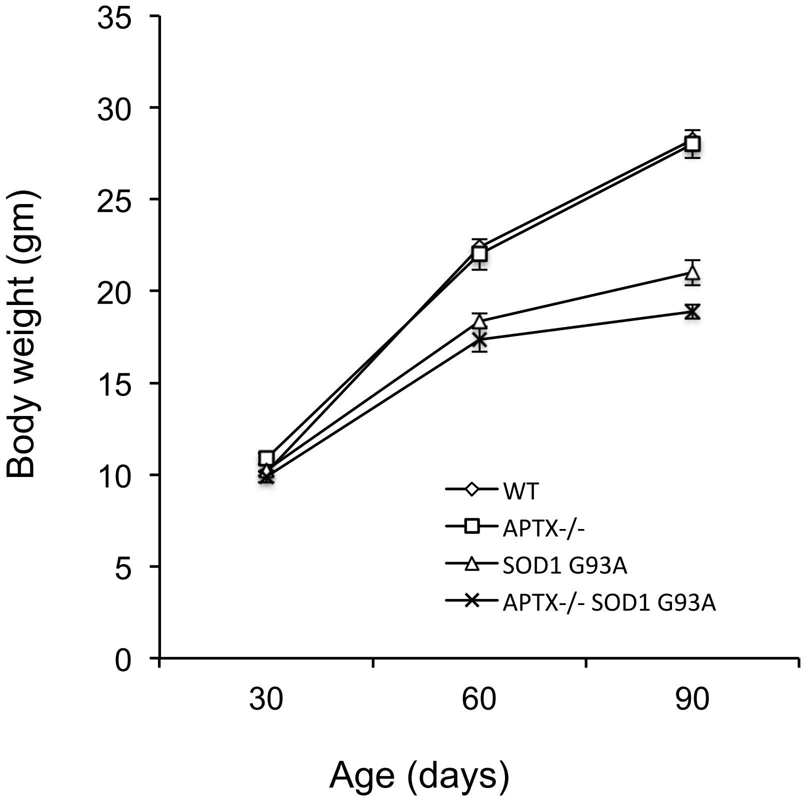

Supplementary figure 2. Body weight of littermate mice with the indicated genetic background (n=7).
